# Disease burden of stroke and its subtypes attributable to low dietary fiber in China, 1990–2019

**DOI:** 10.1038/s41598-024-66639-0

**Published:** 2024-07-09

**Authors:** Shuai Jin, Lang Xie, Junwen Wang, Kaide Xia, Haiwang Zhang

**Affiliations:** 1 School of Biology & Engineering (School of Health Medicine Modern Industry), Guizhou Medical University, No. 6 Ankang Road, Guian New District, Guiyang, 561113 China; 2 Hospital Infection and Preventive Health Care, Bijie Hospital of Zhejiang Provincial People’s Hospital, Bijie, 551700 China; 3 Department of Physical and Mental Diseases, The Second People’s Hospital of Guiyang, No. 547 Jinyang South Road, Guiyang, 550023 China; 4 Guiyang Maternal and Child Health Care Hospital, Guiyang Children’s Hospital, No.63 Ruijin South Road, Guiyang, 550003 China; 5 Department of Neurosurgery, Guizhou Provincial People’s Hospital, Nanming District, No.83, Zhongshan East Road, Guiyang, 550002 China

**Keywords:** Stroke, Low dietary fiber, DALYs, Global burden of disease, Subtypes, Neurology, Risk factors

## Abstract

This study aimed to assess the current status and changing trends of the disease burden of stroke and its subtypes due to low dietary fiber intake in China from 1990 to 2019. In cases of stroke and its subtypes attributable to low dietary fiber, deaths, disability-adjusted life-years (DALYs), age-standardized mortality rates (ASMR), age-standardized DALYs rates (ASDR), and percentage change were used to assess disease burden. Data were obtained from the 2019 global burden of disease study. Trends were assessed using Joinpoint regression and age-period-cohort analysis. Between 1990 and 2019, there was a declining trend in stroke and its subtypes, ASDR and ASMR, as well as the corresponding number of deaths and DALYs, due to low dietary fiber intake in China. Subarachnoid hemorrhage (SH) showed the greatest decrease, followed by intracerebral hemorrhage (IH) and ischemic stroke (IS). Local drift curves showed a U-shaped distribution of stroke, IS, and IH DALYs across the whole group and sex-based groups. For mortality, the overall and male trends were similar to those for DALYs, whereas female stroke, IH, and IS showed an upward trend. The DALYs for stroke and IH showed a clear bimodal distribution, IS showed an increasing risk with age. For mortality, the SH subtype showed a decreasing trend, whereas other subtypes showed an increasing risk with age. Both the period and cohort rates of stroke DALYs and motality due to low dietary fiber have declined. Males had a higher risk of DALYs and mortality associated with low fiber levels. The burden of stroke and its subtypes associated with a low-fiber diet in China has been declining over the past 30 years, with different patterns of change for different stroke subtypes and a higher burden for males, highlighting the differential impact of fiber intake on stroke and its subtypes.

## Introduction

Stroke is the second leading cause of death globally after heart disease and the third leading cause of death and disability, imposing enormous costs on healthcare systems worldwide^1,2^. Since 2005, stroke has been a leading cause of death in China, accounting for approximately one-third of all stroke-related deaths worldwide^3^. The overall lifetime risk of stroke in the Chinese population is 39.3%, which is 1.6 times higher than the average risk in other countries/regions^4^. According to the Global Burden of Disease (GBD) 2019, China’s stroke incidence, prevalence, deaths, and disability-adjusted life-years (DALYs) have shown a sharp upward trend and have become important public health challenges^5^.

A high daily fiber intake is significantly associated with a lower stroke risk^6^. Approximately 90% of strokes can be attributed to variable risk factors, such as environmental^7^, dietary^8^, physiological factors^2^, and economic circumstances^9^. For every dollar invested in stroke and cardiovascular disease prevention, a return of $10.90 is expected^10^. Implementing effective prevention strategies may reduce the stroke burden; however, the effects of low dietary fiber intake on stroke and its subtypes, including ischemic stroke (IS), intracerebral hemorrhage (IH), and subarachnoid hemorrhage (SH), are currently unknown. Therefore, it is important to assess the trends in the disease burden on dietary intake in patients with low-fiber-induced stroke and its subtypes.

This study aimed to explore and compare temporal trends in the disease burden of stroke and subtypes due to low dietary fiber intake in China from 1990 to 2019 using Joinpoint regression and the age-period-cohort method to assess GBD 2019 data.

## Methods

### Study design

All data were obtained from GBD 2019, which provides a comprehensive description of disabilities and deaths in different countries, ages, and genders during the period 1990–2019 and, provides a tool to quantify the burden of a wide range of diseases, injuries, and risk factors (http://ghdx.healthdat.sgbd-results-tool). This study used the tool to obtain the disease burden attributable to low dietary fiber intake for stroke and its subtypes in China from January 1, 1990, to December 31, 2019. The data consisted of the number of deaths, DALYs, age-standardized mortality rate (ASMR), and age-standardized DALYs rate (ASDR). Participants were categorized into age groups of 25–94 years old and 95 years old and above, totaling 15 groups.

### Definitions

Stroke was identified by the swift emergence of clinical indicators, suggesting localized or widespread disruption of brain function that persisted for over 24 h or resulted in fatality. IS is defined as an event of neurological malfunction triggered by a localized infarction of the brain, spinal cord, or retina. IH is a non-traumatic stroke characterized by the localized accumulation of blood in the brain. SH is defined as a stroke resulting from a subarachnoid hemorrhage in the brain that is not caused by trauma^5^.

Age-standardized rate (ASR) is defined as the rate calculated according to the age composition of a standard population. The ASR provides the ability to remove the effects of the population age structure and allows for more accurate comparisons of the mortality and DALYs rate over time. The 2019 ASR was standardized using a global age structure.

The dietary fiber data used in GBD 2019 were mainly derived from nutritional surveys, food frequency questionnaires, and the food and agriculture organization of the United Nations.

The GBD 2019 defines diet low in fiber as an average daily intake < 21–22 g/day from all sources, including fruits, vegetables, grains, and beans, and confirms that there is sufficient evidence of a causal relationship between a diet low in fiber and stroke^11^.

### Statistical analysis

DALYs and deaths attributable to the dietary intake of low fiber and ASRs were estimated using 95% uncertainty intervals (UIs). Joinpoint regression was used to assess the temporal trends in ASMRs and ASDRs for strokes and their subtypes attributable to a low-fiber intake from 1990 to 2019. The model begins by fitting the original data with the least number of knots (e.g., 0 knots, indicating a straight line). It then assesses whether additional knots are statistically significant and need to be included. The limit on the number of knots is influenced by the input data. In this study, given that the number of observations is at least 29, the maximum number of knots is set to 5 by default, which divides the data into 6 trend groups to ensure robust results. Long-term trends in both metrics were divided into segments and statistically significant trends at different time periods were identified. The average annual percentage change (AAPC), annual percentages change (APC) for each segment, and their 95% confidence intervals (95% CIs) indicated the direction and magnitude of the trends. The APC describes the average annual rate of change in a burden variable over a specific time period. The AAPC summarizes the overall trend for the entire study period by averaging the APCs across multiple segments. Age-period-cohort analysis assessed the contribution of a low-fiber diet to the age, period, and cohort effects of stroke and its subtypes of death and DALYs. Decomposition of these three trends provided relatively valid estimates. The age effect refers to the physiological and social processes of aging. The period effect represents changes over time in the burden of stroke disease and subtypes attributable to diet in low-fiber groups that are simultaneously associated with all age groups. The cohort effect is the change in a subject cohort over time due to different risk factors and tubing exposures. Local differences in the AAPC for each age group were determined after adjusting for period and birth cohort. Longitudinal age curves represent the longitudinal age-specific disease burden after adjusting for cohort and period. We estimated local drifts, longitudinal age curves, and period and cohort rate ratios (RRs).

### Ethics statement

The GBD database is publicly available and does not contain patient information; consequently, ethical review and approval was waived.

## Results

**Figure 1:**
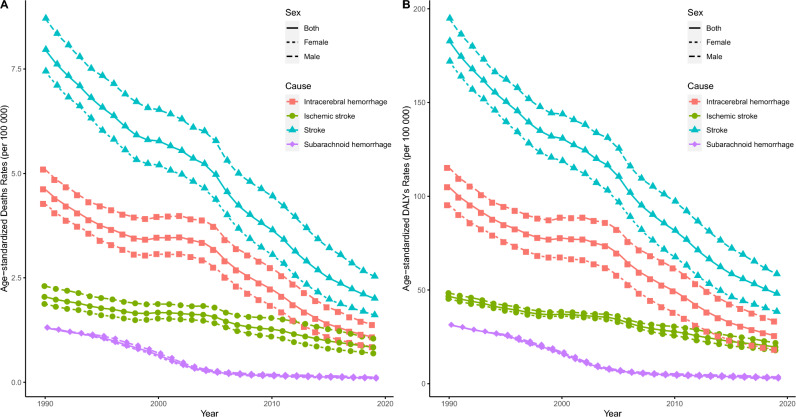
Trends in ASDR (**A**) and ASMR (**B**) of stroke and its subtypes attributable to low dietary fiber from 1990 to 2019. *ASDR* age-standardized disability-adjusted life-years rates, *ASMR* age-standardized mortality rates.

Table 1, Fig. 1, Table S1 and Figures S1–S3 present the trends in the disease burden of stroke and its subtypes, including mortality and DALYs, attributed to a low-fiber diet. In 2019, 35,300 stroke deaths were attributed to low dietary fiber in China. Regarding the subtypes of stroke, there were 19,400, 1900, and 14,100 patient deaths in the IH, SH and IS groups. Furthermore, male deaths were higher than those of females. In terms of DALYs, nearly 930,000 cases were attributed to low dietary fiber intake, with a distribution of 494,100, 60,050, and 371,000 for the IH, SH, and IS subtypes, respectively. Men had more DALYs than women. ASDR and ASMR showed the same trend as the case distribution. From 1990 to 2019, the ASMR and ASDR for stroke and its subtypes showed similar decreasing trends, with stroke showing the largest decrease, followed by IH, IS, and SH. There was little difference in the magnitude of decline in ASMR and ASDR between males and females in the SH subtype. In stroke and other subtypes, the magnitude of decline was higher in males than in females. The overwhelming majority of Chinese strokes and their subtypes attributable to a low-fiber diet showed a downward trend in the number of deaths, DALYs, and ASRs, with a non-significant case and ASRs percent change in IS. Among all sexes, SH showed the greatest decrease in ASMR, followed by IH and IS. Joinpoint regression analyses revealed a decreasing trend in stroke ASMR and ASDR attributable to low-fiber intake in China. For ASMR, the largest decrease in total gender SH was observed (AAPC: −8.45 [95% CI −8.75 to −8.15]), followed by IH and IS (−4.88 [95% CI −5.13 to −4.62], and −3.02 [95% CI −3.26 to −2.78], respectively). All stroke ASMR attributable to low dietary fiber intake declined the most between 2010 and 2015; SH declined the most, with a decline of 19.16% between 2000 and 2004. The ASDR also showed a decreasing trend, with the largest decrease in stroke ASDR of −6.87 occurring between 2004 and 2007. Among the subtypes, SH showed a higher rate of decline. The trend was similar between sexes; however, the rate of decline was higher in females.

**Table 1:** Number and age-standardized rates of deaths and DALYs due to stroke in 2019, and the percentage change in China from 1990 to 2019, stratified by pathological types of stroke.

	Stroke		Intracerebral hemorrhage		Subarachnoid hemorrhage		Ischemic stroke	
	Number, 10000	ASR, per 100000	Number, 10000	ASR, per 100000	Number, 10000	ASR, per 100000	Number, 10000	ASR, per 100000
Both								
Deaths								
2019	3.53(0.90, 7.17)	2.01(0.52, 4.08)	1.94(0.47, 3.96)	1.07(0.27, 2.2)	0.19(0.04, 0.39)	0.1(0.02, 0.21)	1.41(0.38, 2.88)	0.83(0.23, 1.7)
Percentage change (%), 1990–2019	−0.37(−0.57, −0.11)	−0.75(−0.83, −0.65)	−0.41(−0.6, −0.18)	−0.77(−0.84, −0.67)	−0.81(−0.89, −0.64)	−0.92(−0.95, −0.85)	0.09(−0.3, 0.57)	−0.59(−0.74, −0.42)
AAPC, 1990–2019		−4.62(−4.87, −4.38)*		−4.88(−5.13, −4.62)*		−8.45(−8.75, −8.15)*		−3.02(−3.26, −2.78)*
DALYs								
2019	92.57(22.05, 188.41)	48.14(11.63, 97)	49.41(11.55, 102.50)	25.43(5.91, 51.63)	6.05(1.39, 12.50)	3.15(0.72, 6.48)	37.10(9.41, 74.62)	19.55(5.02, 39.18)
Percentage change (%), 1990–2019	−0.42(−0.61, −0.21)	−0.74(−0.82, −0.65)	−0.47(−0.65, −0.26)	−0.76(−0.84, −0.67)	−0.79(−0.87, −0.61)	−0.9(−0.94, −0.82)	−0.05(−0.35,0.3)	−0.58(−0.7, −0.43)
AAPC, 1990–2019		−4.47(−4.73, −4.22)*		−4.74(−4.98, −4.5)*		−7.6(−7.88, −7.32)*		−2.93(−3.17, −2.69)*
Male								
Deaths								
2019	1.98(0.50, 4.14)	2.54(0.66, 5.27)	1.12(0.27, 2.36)	1.37(0.35, 2.9)	0.10(0.02, 0.23)	0.12(0.03, 0.27)	0.76(0.20, 1.60)	1.05(0.29, 2.21)
Percentage change (%), 1990–2019	−0.3(−0.57, 0.08)	−0.71(−0.81, −0.56)	−0.34(−0.61, 0.01)	−0.73(−0.83, −0.6)	−0.78(−0.88, −0.16)	−0.91(−0.95, −0.65)	0.16(−0.32, 0.83)	−0.55(−0.72, −0.29)
AAPC, 1990–2019		−4.17(−4.47, −3.87)*		−4.44(−4.79, −4.08)*		−7.86(−8.11, −7.61)*		−2.67(−2.87, −2.46)*
DALYs								
2019	54.15(1.249, 114.13)	58.61(14.12, 121.99)	31.18(6.94, 65.49)	33.18(7.56, 69.83)	3.45(0.77, 7.55)	3.68(0.84, 8.01)	19.52(4.86, 41.37)	21.75(5.62, 45.35)
Percentage change (%), 1990–2019	−0.36(−0.62, −0.04)	−0.7(−0.81, −0.55)	−0.39(−0.64, −0.06)	−0.71(−0.83, −0.57)	−0.76(−0.87, −0.24)	−0.88(−0.94, −0.63)	0(−0.4, 0.5)	−0.55(−0.72, −0.34)
AAPC, 1990–2019		−4.06(−4.36, −3.76)*		−4.2(−4.61, −3.78)*		−7.13(−7.33, −6.92)*		−2.69(−2.89, −2.49)*
Female								
Deaths								
2019	1.55(0.38, 3.33)	1.61(0.39, 3.41)	0.82(0.19, 1.78)	0.84(0.2, 1.8)	0.08(0.02, 0.17)	0.08(0.02, 0.17)	0.65(0.16, 1.39)	0.69(0.17, 1.46)
Percentage change (%), 1990–2019	−0.44(−0.67, −0.14)	−0.78(−0.87, −0.67)	−0.49(−0.7, −0.22)	−0.8(−0.88, −0.7)	−0.84(−0.91, −0.73)	−0.94(−0.96, −0.9)	0.01(−0.41, 0.57)	−0.63(−0.78, −0.43)
AAPC, 1990–2019		−5.14(−5.42, −4.85)*		−5.43(−5.75, −5.1)*		−9.05(−9.31, −8.79)*		−3.38(−3.7, −3.05)*
DALYs								
2019	38.42(9.31, 80.44)	38.51(9.37, 79.72)	18.24(4.26, 39.26)	18.07(4.27, 38.46)	2.60(0.59, 5.23)	2.66(0.61, 5.34)	17.58(4.46, 36.65)	17.78(4.54, 36.84)
Percentage change (%), 1990–2019	−0.49(−0.68, −0.27)	−0.78(−0.86, −0.68)	−0.57(−0.74, −0.36)	−0.81(−0.88, −0.73)	−0.82(−0.89, −0.71)	−0.92(-0.95, −0.87)	−0.1(−0.42, 0.29)	−0.61(−0.74, −0.45)
AAPC, 1990–2019		−5.01(−5.31, −4.72)*		−5.52(−5.83, −5.21)*		−8.18(−8.44, −7.93)*		−3.15(−3.46, −2.83)*

*AAPC* average annual percentage change.

*implicates p < 0.05.

**Figure 2:**
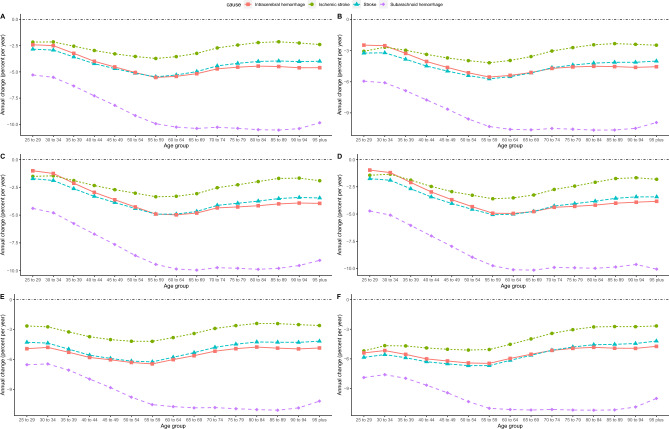
Local drift for stroke and its subtypes DALYs  and mortality attributable to low dietary fiber in China. (**A**) All DALYs, (**B**) male DALYs, (**C**) female DALYs, (**D**) all mortality, (**E**) male mortality, and (**F**) female mortality. *DALYs* disability-adjusted life-years.

We calculated the local drifts using age-period-cohort analysis, which was used to assess the AAPC of ASMR and ASDR for stroke attributable to a low-fiber diet across age groups in China (Fig. 2). After controlling for period and cohort effects, the local drift curves of ASDR for stroke in different age groups, except SH, showed a U-shape, with the largest decline in age 55–59 years, a general downward trend in stroke and IH, and leveling off in SH after a sharp decline before the age group 65–69 years. The local drift curves of stroke and its subtypes in ASMR and ASDR showed the same trend. From the male perspective, the local drift curves of the ASDR and ASMR coincided with the trend of the curves for the entire population. The local drift curves of ASDR in females showed a U-shaped distribution, except for SH, which showed the same trend as the entire population and males. The local drift curves of stroke, SH, and IS in female ASMR showed an increasing trend, whereas SH showed a decreasing trend. In all age groups, SH mortality decreased more than IS and ICH mortality regardless of sex. Additionally, females showed a greater decrease than males in both stroke subtypes and age groups, as well as in ASDR and ASMR.

**Figure 3:**
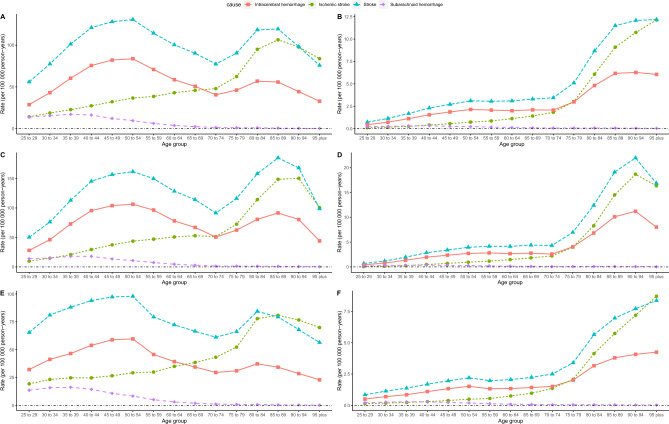
Longitudinal age curves of low dietary fiber attributable stroke and its subtype DALYs and mortality in China. (**A**) All DALYs, (**B**) male DALYs, (**C**) female DALYs, (**D**) all motality, (**E**) male mortality, and (**F**) female mortality. *DALYs* disability-adjusted life-years.

The longitudinal age profiles of stroke and its subtypes, DALYs and mortality, according to low dietary fiber intake in China are shown in Fig. 3. We observed a bimodal distribution of IH and stroke DALYs with age in both sexes. Regardless of the overall and differentiated male and female sex, the highest DALYs was found in the stroke and IH patient age group 50–54 years, which was the highest value of the first peak. The DALYs was also higher in the stroke and IH patient age group 80–89 years. The IS DALYs increased significantly with age, especially in patients > 75 years. SH DALYs showed a decreasing trend with age, with lower DALYs in patients > 70 years. In terms of mortality, stroke, IH, and IS showed an increasing trend with age, especially > 70 years, when the slope of increase was steeper. After 75 years of age, the mortality of the stroke and IS groups was higher than that of the IH group. However, mortality of the SH showed a decreasing trend with age. The DALYs and mortality RRs were much higher in males in all age groups.

**Figure 4:**
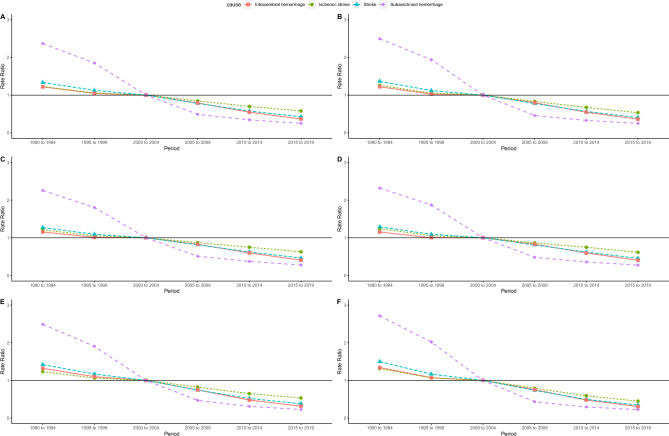
Period rate ratio (RR) of low dietary fiber attributable stroke and its subtypes DALYs and mortality in China. (**A**) All DALYs, (**B**) male DALYs, (**C**) female DALYs, (**D**) all mortality, (**E**) male mortality, and (**F**) female mortality. *DALYs* disability-adjusted life-years.

Figures 4 and S4 show the estimated period and cohort effects of stroke attributable to low fiber dietary intake and its subtypes DALYs and mortality in China. The results showed that from 1990 to 2019, the DALYs and risk of death from stroke attributable to low-fiber diet generally trended downward, with the steepest slope for SH and the flattest slope for IS. Overall, the RR for the SH was higher before 2000 and lower after 2004 compared with 2000–2004. In contrast, stroke, IS, and IH showed linearly decreasing trends. The trends of DALYs and mortality were similar overall and across sexes. Cohort effects showed that SH mortality and DALYs had the steepest overall and single-sex slopes and the fastest rates of decline, followed by stroke and IH. The rate of decline was higher before 1955 than after 1964. The IS showed a linear downward trend, which was not significantly different from that before and after 1955–1964. Regardless of the DALYs or mortality, the downward trend in cohort effects was more pronounced in women.

## Discussion

This study found a significant downward trend in the ASDRs and ASMRs for stroke due to low dietary fiber intake among Chinese residents from 1990 to 2019. The disease burden of all stroke subtypes, including the AAPCs, periods, and cohort RRs of ASMRs and ADSRs, showed a significant downward trend, with SH showing the largest decline, and IS the smallest. Notably, the DALYs associated with a low-fiber diet showed a bimodal trend with age in the stroke and IH subtypes, whereas the DALYs in the IS increased progressively with age and the opposite trend was observed in SH. The SH and IS mortality attributable to low dietary fiber intake increased significantly with age. Across all stroke subtypes, the disease burden was higher in men, and the decline was smaller.

Fiber is an essential component of the diet and plays a multifaceted role in the prevention and treatment of stroke. Multiple meta-analyses have shown that there is a significant negative dose-response relationship between dietary fiber intake and stroke risk, and that eating foods rich in fiber may prevent incident stroke^6,12–16^. Two American population-based National Health and Nutrition Examination Survey studies^17,18^ and a cohort study of postmenopausal women in the United States^19^ confirmed that dietary fiber intake is an important factor influencing stroke; higher dietary fiber intake was associated with a lower incidence of stroke. A longitudinal nutritional study in Jiangsu Province, China, confirmed the benefits of dietary fiber from whole grain products, vegetables, and fruits in preventing the development of chronic comorbidities, including stroke, in China^20^. Casiglia et al. reported that stroke incidence was reduced by 50 or 46% when daily soluble and insoluble fiber intake was > 25 g and > 47 g, respectively; higher dietary fiber intake was negatively and independently associated with stroke incidence and risk^21^. The Japanese diet and specialty Japanese foods may reduce mortality from cardiovascular disease^15^. Recommendations for healthy behaviors for the primary prevention of stroke in recent guidelines are almost identical, including weight loss, reduced salt intake, and increased fruit and vegetable intake. Fruits and vegetables contain high levels of potassium, antioxidants, phytochemicals, and dietary fiber; therefore, reduce the incidence of cardiovascular disease and its risk factors^22^. It is widely believed that the consumption of a high-fiber diet promotes bacterial diversity, increasing beneficial bacteria while decreasing potentially pathogenic bacteria, and thus, the gut microbiota, as well as immune and inflammatory markers^23^. Therefore, a high-fiber diet may stimulate the production of anti-inflammatory cytokines in the gut and reduce post-stroke brain damage. In a study of 178 older adults, Claesson et al. reported that people who consumed a high-fiber diet produced more butyrate and acetate from short-chain fatty acids, thereby improving stroke prognosis^24^. Similar conclusions were reported in studies conducted in mice^25^. Fiber intake plays an important role in post-stroke nutritional interventions^26^. High oat fiber intake is associated with fewer future stroke events in patients with coronary heart disease undergoing secondary prevention after coronary intervention^27^. Plant-based, low-fat, high-fiber diets rich in antioxidants and other lifestyle interventions can reduce the burden and disability associated with common neurological disorders. Dietary interventions may potentially influence the pathophysiological processes of neurological disorders, favorably altering clinical outcomes. Appropriate dietary choices should be viewed as a part of a continuum of healthy lifestyle choices^28^. Except for a few countries or territories, the burden of disease attributable to low-fiber diets has been declining globally, but changes within the study period and estimates of effects are less clear^29,30^. Joinpoint regression and age-period-cohort analysis, which look at trends in burden indicators within the study period and estimate age-period and cohort effects, have significant advantages for assessing disease burden. They also estimate age, period, and cohort effects, and have significant advantages for assessing disease burden^31^. Therefore, the present study comprehensively investigated the disease burden of stroke and its subtypes due to low dietary fiber intake using the GBD database.

Overall, the burden of stroke and its subtypes attributable to a low-fiber diet has declined in China over the past 30 years, as evidenced by decreases in ASDRs and ASMRs, and corresponding decreases in deaths and DALYs. Notably, ASDRs, ASMRs, and RRs attributable to a low-fiber diet showed the greatest decline in the SH group. The decline in the SH disease burden is more likely due to advances in treatment than to the amount of dietary fiber intake, as the increase in advanced neurovascular imaging and endovascular interventions has halved the mortality rate in SH^32^. Additionally, the burden of IS attributable to a low-fiber diet showed a decreasing trend, which may be related to the increased use of intravenous thrombolysis. Studies have confirmed that stroke mortality rates in China differ between urban and rural areas/regions, with rural patients having higher stroke mortality rates than urban patients, and Southwest China having higher mortality rates than other regions^33^. This may be related to the level of the economy, utilization of healthcare resources, and perception of the population. The high mortality rate of rural patients may result from the lack of timely treatment due to the distance from the healthcare facility, and areas with better economic development may have a greater focus on nutritional balance. A national platform for remote stroke centers was launched in 2014, and a network is currently being developed to guide timely stroke treatment in rural hospitals^34^. It is encouraging that a low-fiber diet has been declining in recent years^11,35^. Compared to GBD 2017, GBD 2019 showed a significant improvement in the proportion of dietary fiber leading to a poor stroke prognosis. However, one of the main reasons for this result is that the GBD 2019 adopts a more relaxed definition of a low-fiber diet. A daily intake of 25–29 g of dietary fiber is currently considered an appropriate and achievable goal; however, the actual intake is well below the target. In the United Kingdom, the recommended daily intake has increased to 30 g; however, only 13% of men and 4% of women meet this recommendation^36^. A 2015 survey showed that only 2.5% of the Chinese population met the minimum recommended daily intake of fiber, with a total intake of only 9.7 g/person/day^37^. Cereals are the main source of dietary fiber in China; however, since 1982, cereal consumption in China has continued to decline, whereas refined grain consumption has increased^38^. The main reason for this phenomenon is rapid economic development and changing dietary patterns, whereby low-income countries are reducing their intake of plant-based foods in favor of animal and processed foods^39,40^. Although the burden of stroke in China attributable to dietary intake of low fiber is decreasing, it should not be ignored. In the future, it will be necessary to improve the dietary structure in China and increase dietary fiber intake to reduce the risk of stroke^41^.

Additionally, different sources of fiber have different effects on the prognosis of stroke and its subtypes. A study of nearly 370,000 people in the United States confirmed a 15–34% reduction in the risk of disease-specific deaths, including stroke, among those with the highest cereal fiber intake. After further adjustment for grain fiber, the association with whole-grain stroke was no longer significant^42^. Much of the current research has focused on the effects of dietary fiber sources on the incidence of stroke and its subtypes. Higher total dietary, fruit, vegetable, soluble, and insoluble fiber levels have positive effects on stroke risk reduction. However, grain fibers did not significantly reduce the risk of stroke. For different stroke types, increased total dietary fiber was associated with ischemic stroke with a positive effect but was not found in hemorrhagic stroke. Stroke risk decreased with an increase in total dietary fiber intake. Increased dietary fiber intake has a positive effect on reducing stroke risk, and different dietary fibers have different effects on stroke subtypes^43^. A Swedish study found that a high intake of total fiber and fiber from fruits and vegetables, but not from grains, was negatively associated with the risk of stroke. After adjusting for other stroke risk factors, the multivariate relative hazard ratios for total stroke were 0.90, for total fiber 0.85, and for fruit fiber 0.90. These findings suggest that the intake of dietary fibers, especially fruit and vegetable fibers, is negatively associated with stroke risk^44^. Similar conclusions were obtained in another study, where dietary fiber intake had a significant protective effect against ischemic stroke but not hemorrhagic stroke^45,46^. An American study found that only higher sources of viscous fiber and phytosterol intake were associated with a lower risk of stroke^47^. A British study found that among women of healthy weight, more grain fiber was associated with a lower risk of ischemic stroke, and higher fiber density was associated with a lower risk of ischemic stroke^48^. A study of male smokers found that vegetable consumption was negatively associated with the risk of subarachnoid hemorrhage, and grain consumption was negatively associated with the risk of intracerebral hemorrhage^49^. This is probably because grain fiber intake is associated with lower levels of various markers of inflammation and a lower risk of cardiovascular disease; additionally, inflammation mediates one-sixth of the relationship between grain fiber intake and cardiovascular disease^50^. In any event, it is recommended to increase dietary fiber intake to prevent stroke.

The present study had several limitations. First, the dietary fiber intake of Chinese residents was estimated using dietary questionnaires, which may introduce bias that affects the validity of dietary fiber intake estimates. Second, the GBD did not classify the sources of dietary fiber, and the effects of different fiber sources on stroke and its subtypes were inconsistent. Third, the GBD did not classify data related to Chinese provinces, and the burden of diseases attributable to a low-fiber diet could not be assessed across Chinese provinces. Fourth, by analyzing stroke and its subtypes of death and DALYs attributable to low-fiber intake in the Chinese population from a global perspective, this study only highlighted the benefits of increasing fiber intake at the national level, which may not be instructive for individuals. In addition, the type of dietary fiber may influence the burden of stroke and its subtypes, but more profound studies have not been done in the database. Finally, other determinants may also confound the association between low fiber intake and stroke and its subtypes, including lifestyle, medication use, and risk factor management. Therefore, further studies are warranted.

Our study provides strong evidence for assessing the disease burden of stroke and its subtypes attributable to the low dietary fiber intake in China. This stroke burden has been declining over the past 30 years, with different patterns of change in different stroke subtypes. This suggests that dietary fiber intake should be emphasized for stroke prevention in China, especially in elderly individuals and men.

### Supplementary Information

Supplementary Information.

## Data Availability

The data used in this study can be downloaded from the GBD database or obtained from the corresponding author.

## Abbreviations

*AAPC*: Average annual percentage change
*ASMR*: Age-standardized mortality rate
*ASR*: Age-standardized rate
*ASDR*: Age-standardized disability-adjusted life-years rate
*CI*: Confidence interval
*DALYs*: Disability-adjusted life-years
*GBD*: Global burden of disease
*IH*: Intracerebral hemorrhage
*IS*: Ischemic stroke
*RRs*: Rate ratios
*SH*: Subarachnoid hemorrhage
*UIs*: Uncertainty intervals
