# Supplementary material for: Disease burden of stroke and its subtypes attributable to low dietary fiber in China, 1990–2019
Source: Sci Rep. 2024 Jul 9;14:15854. doi: 10.1038/s41598-024-66639-0 (PMC11233718; doi:10.1038/s41598-024-66639-0)
Supplement: Supplementary file 1 — Supplementary Information. [file 41598_2024_66639_MOESM1_ESM.docx]

**Disease Burden of Stroke and Its Subtypes Attributable to Low Dietary Fiber in China, 1990–2019**

**Shuai Jin^1†^, Lang Xie^2†^, Junwen Wang^3^, Kaide Xia^4*†^, Haiwang Zhang^5*†^**

^1^School of Biology & Engineering (School of Health Medicine Modern Industry), Guizhou Medical University, No. 6 Ankang Road, Guian New District 561113, China

^2^Department of Preventive Health Care, Bijie Hospital of Zhejiang Provincial People's Hospital, Bijie 551700, China

^3^Department of Physical and Mental Diseases, The Second People's Hospital of Guiyang, No. 547 Jinyang South Road 550023, Guiyang, China.

^4^Guiyang Maternal and Child Health Care Hospital, Guiyang Children's Hospital, No.63 Ruijin South Road 550003, Guiyang, China.

^5^Department of Neurosurgery, Guizhou Provincial People's Hospital, No.83, Zhongshan East Road, Guiyang 550002, Guiyang, China

^†^ These authors contributed equally to this work.

**^*^ Correspondence to:**

**Haiwang Zhang,** post-MD, Department of Neurosurgery, Guizhou Provincial People's Hospital, No.83, Zhongshan East Road, Nanming District, Guiyang 550002, China. Telephone +86 17365013004, Email [zhw_cx@163.com](mailto:zhw_cx@163.com).

**Kaide Xia**, Guiyang Maternal and Child Health Care Hospital, Guiyang Children's Hospital, No.63 Ruijin South Road 550003, Guiyang, China. Telephone +86 18185021587, Email xiakaide@126.com.

*Drs Jin and Xie are joint first authors.

| Table S1 Joinpoint regression analysis of the disease burden attributable to low dietary fiber for stroke and its subtypes in China. | | | | | | | |
| --- | --- | --- | --- | --- | --- | --- | --- |
|  | Segment | Both | | Male | | Female | |
|  |  | Period | APC (95% CI) | Period | APC (95% CI) | Period | APC (95% CI) |
| Deaths |  |  |  |  |  |  |  |
| Stroke | 1 | 1990-1998 | -3.53(-3.69, -3.36) | 1990-1997 | -3.24(-3.5, -2.98) | 1990-1998 | -3.99(-4.18, -3.8) |
|  | 2 | 1998-2004 | -1.86(-2.21, -1.5) | 1997-2004 | -1.78(-2.13, -1.43) | 1998-2004 | -2.09(-2.49, -1.68) |
|  | 3 | 2004-2007 | -7.22(-8.73, -5.7) | 2004-2007 | -6.14(-8.13, -4.11) | 2004-2007 | -8.03(-9.76, -6.27) |
|  | 4 | 2007-2010 | -4.79(-6.34, -3.21) | 2007-2010 | -3.69(-5.76, -1.58) | 2007-2010 | -5.96(-7.74, -4.14) |
|  | 5 | 2010-2015 | -7.35(-7.84, -6.86) | 2010-2019 | -6.19(-6.39, -6) | 2010-2015 | -8.33(-8.89, -7.77) |
|  | 6 | 2015-2019 | -5.33(-5.85, -4.8) | … | … | 2015-2019 | -5.02(-5.64, -4.4) |
| Intracerebral  hemorrhage | 1 | 1990-1997 | -3.76(-4.1, -3.41) | 1990-1994 | -4.07(-4.96, -3.17) | 1990-1998 | -4.09(-4.31, -3.87) |
|  | 2 | 1997-2004 | -0.49(-0.94, -0.03) | 1994-1998 | -2.07(-3.56, -0.55) | 1998-2001 | 0.64(-1.46, 2.77) |
|  | 3 | 2004-2011 | -6.94(-7.36, -6.51) | 1998-2004 | -0.22(-0.91, 0.47) | 2001-2004 | -1.48(-3.56, 0.64) |
|  | 4 | 2011-2015 | -9.31(-10.57, -8.05) | 2004-2011 | -5.93(-6.43, -5.42) | 2004-2011 | -7.84(-8.18, -7.5) |
|  | 5 | 2015-2019 | -6.15(-7.01, -5.27) | 2011-2015 | -8.4(-9.86, -6.91) | 2011-2015 | -10.45(-11.46, -9.43) |
|  | 6 | … | … | 2015-2019 | -6.61(-7.6, -5.61) | 2015-2019 | -5.95(-6.65, -5.23) |
| Subarachnoid  hemorrhage | 1 | 1990-1996 | -4.18(-4.57, -3.8) | 1990-1996 | -3.45(-3.76, -3.15) | 1990-1995 | -4.07(-4.45, -3.7) |
|  | 2 | 1996-2000 | -10.22(-11.3, -9.13) | 1996-2000 | -9.5(-10.36, -8.63) | 1995-2000 | -10.06(-10.56, -9.57) |
|  | 3 | 2000-2004 | -19.16(-20.12, -18.19) | 2000-2004 | -18.54(-19.28, -17.79) | 2000-2004 | -19.87(-20.56, -19.17) |
|  | 4 | 2004-2007 | -13.06(-15.13, -10.94) | 2004-2007 | -11.93(-13.53, -10.29) | 2004-2007 | -13.56(-15.1, -12) |
|  | 5 | 2007-2019 | -4.89(-5.04, -4.75) | 2007-2016 | -4.09(-4.29, -3.9) | 2007-2010 | -6.87(-8.54, -5.17) |
|  | 6 |  |  | 2016-2019 | -6.03(-6.96, -5.09) | 2010-2019 | -5.15(-5.32, -4.99) |
| Ischemic stroke | 1 | 1990-1998 | -2.54(-2.72, -2.36) | 1990-1998 | -2.46(-2.64, -2.29) | 1990-1997 | -2.95(-3.24, -2.66) |
|  | 2 | 1998-2004 | -0.2(-0.57, 0.18) | 1998-2004 | -0.43(-0.8, -0.05) | 1997-2004 | -0.24(-0.62, 0.14) |
|  | 3 | 2004-2007 | -5.68(-7.27, -4.06) | 2004-2007 | -4.38(-5.98, -2.74) | 2004-2007 | -6.37(-8.5, -4.19) |
|  | 4 | 2007-2011 | -2.84(-3.67, -1.99) | 2007-2012 | -1.73(-2.27, -1.18) | 2007-2011 | -4.06(-5.18, -2.92) |
|  | 5 | 2011-2015 | -5.26(-6.09, -4.42) | 2012-2019 | -4.71(-4.94, -4.48) | 2011-2015 | -6.28(-7.39, -5.16) |
|  | 6 | 2015-2019 | -4.03(-4.59, -3.47) | … | … | 2015-2019 | -3.59(-4.35, -2.83) |
| DALYs |  |  |  |  |  |  |  |
| Stroke | 1 | 1990-1997 | -3.72(-3.94, -3.51) | 1990-1997 | -3.44(-3.71, -3.18) | 1990-1998 | -4(-4.22, -3.78) |
|  | 2 | 1997-2004 | -2.37(-2.65, -2.09) | 1997-2004 | -1.9(-2.25, -1.56) | 1998-2001 | -2.15(-4.14, -0.11) |
|  | 3 | 2004-2007 | -6.87(-8.45, -5.27) | 2004-2007 | -6.05(-8.03, -4.02) | 2001-2004 | -3.71(-5.74, -1.63) |
|  | 4 | 2007-2010 | -4.97(-6.6, -3.3) | 2007-2010 | -4.01(-6.07, -1.91) | 2004-2015 | -7.03(-7.19, -6.87) |
|  | 5 | 2010-2015 | -6.46(-6.99, -5.92) | 2010-2019 | -5.52(-5.72, -5.32) | 2015-2019 | -4.52(-5.23, -3.81) |
|  | 6 | 2015-2019 | -4.71(-5.27, -4.15) | … | … | … | … |
| Intracerebral  hemorrhage | 1 | 1990-1996 | -4.11(-4.5, -3.71) | 1990-1994 | -4.2(-5, -3.39) | 1990-1998 | -4.04(-4.25, -3.83) |
|  | 2 | 1996-2004 | -1.11(-1.44, -0.78) | 1994-1997 | -2.53(-5.2, 0.22) | 1998-2001 | -0.21(-2.18, 1.8) |
|  | 3 | 2004-2011 | -6.71(-7.11, -6.31) | 1997-2004 | -0.53(-1, -0.06) | 2001-2004 | -2.47(-4.44, -0.46) |
|  | 4 | 2011-2015 | -8.27(-9.47, -7.07) | 2004-2011 | -5.64(-6.09, -5.19) | 2004-2011 | -8.08(-8.41, -7.75) |
|  | 5 | 2015-2019 | -5.7(-6.51, -4.88) | 2011-2014 | -7.65(-10.29, -4.94) | 2011-2015 | -9.78(-10.76, -8.78) |
|  | 6 | … | … | 2014-2019 | -6.07(-6.71, -5.42) | 2015-2019 | -5.67(-6.36, -4.97) |
| Subarachnoid  hemorrhage | 1 | 1990-1996 | -4.24(-4.52, -3.95) | 1990-1996 | -3.71(-3.96, -3.46) | 1990-1995 | -4.05(-4.41, -3.69) |
|  | 2 | 1996-2000 | -9.45(-10.26, -8.63) | 1996-2000 | -8.98(-9.68, -8.27) | 1995-2000 | -9.21(-9.69, -8.73) |
|  | 3 | 2000-2004 | -17.01(-17.74, -16.27) | 2000-2004 | -16.57(-17.19, -15.94) | 2000-2004 | -17.61(-18.3, -16.92) |
|  | 4 | 2004-2007 | -10.85(-12.46, -9.21) | 2004-2007 | -10.54(-11.89, -9.17) | 2004-2007 | -11.38(-12.88, -9.86) |
|  | 5 | 2007-2010 | -5.22(-6.97, -3.43) | 2007-2016 | -3.57(-3.74, -3.41) | 2007-2010 | -6.86(-8.51, -5.19) |
|  | 6 | 2010-2019 | -4.18(-4.35, -4.01) | 2016-2019 | -5.01(-5.8, -4.21) | 2010-2019 | -4.72(-4.88, -4.56) |
| Ischemic stroke | 1 | 1990-1998 | -2.76(-2.92, -2.6) | 1990-1998 | -2.76(-2.92, -2.6) | 1990-1998 | -2.84(-3.03, -2.65) |
|  | 2 | 1998-2004 | -0.59(-0.92, -0.25) | 1998-2004 | -0.58(-0.93, -0.24) | 1998-2001 | -0.01(-1.8, 1.82) |
|  | 3 | 2004-2007 | -5.29(-6.72, -3.84) | 2004-2007 | -4.63(-6.11, -3.12) | 2001-2004 | -1.35(-3.18, 0.52) |
|  | 4 | 2007-2011 | -3.16(-3.91, -2.4) | 2007-2011 | -2.02(-2.79, -1.24) | 2004-2007 | -5.36(-7.15, -3.54) |
|  | 5 | 2011-2014 | -4.41(-5.92, -2.88) | 2011-2019 | -3.77(-3.95, -3.6) | 2007-2015 | -4.5(-4.75, -4.25) |
|  | 6 | 2014-2019 | -3.45(-3.8, -3.09) | … | … | 2015-2019 | -3.02(-3.66, -2.39) |

**Supplemental Figures:**


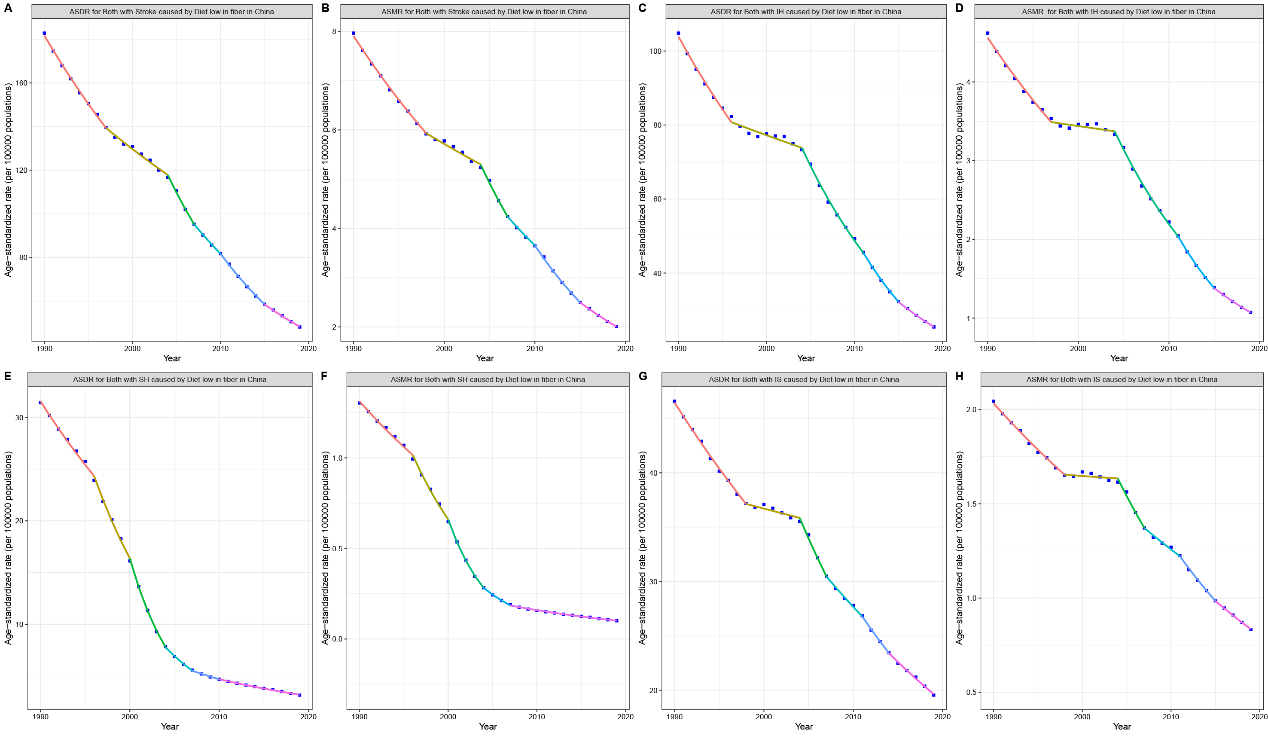


Figure S1. Trends of ASDR and ASMR of Stroke and its Subtypes Attributable to Low Dietary Fiber From 1990 to 2019 in China Using Joinpoint Regression. ASDR: age-standardized disability-adjusted life-years rates; ASMR: age-standardized mortality rates.


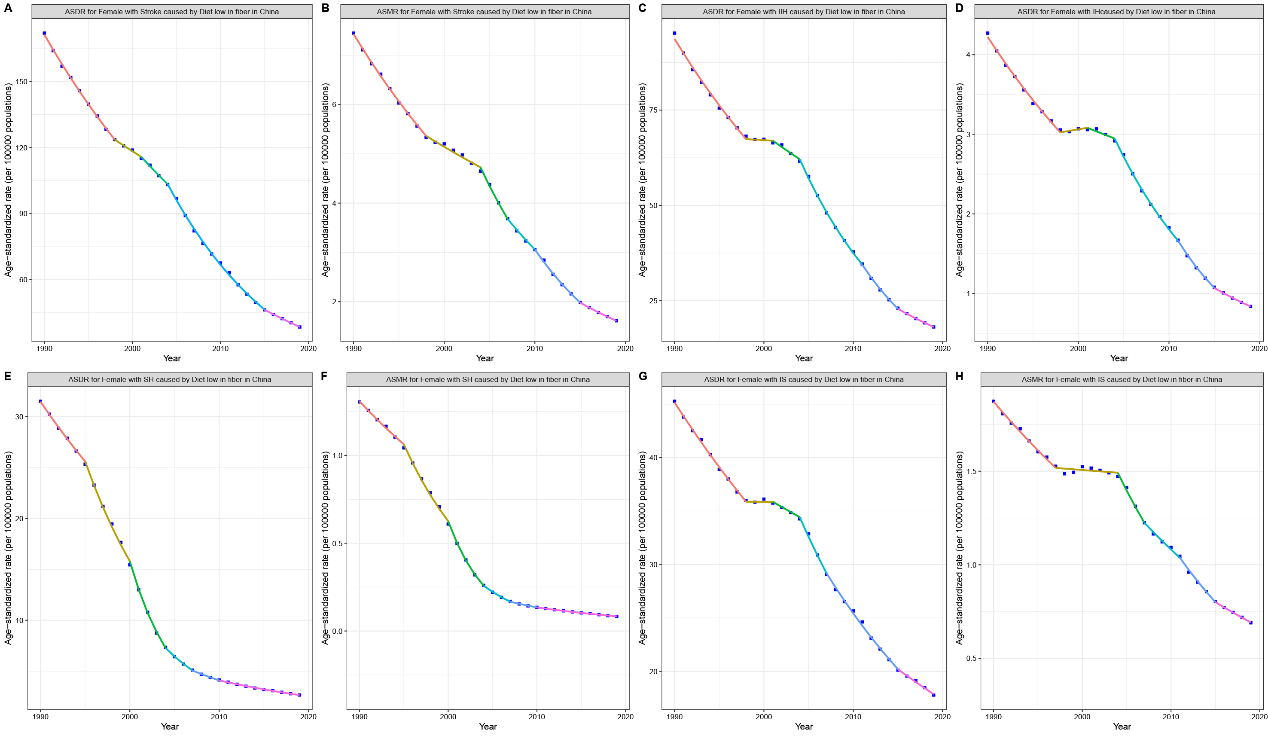


Figure S2. Trends of ASDR and ASMR of Stroke and its Subtypes Attributable to Low Dietary Fiber From 1990 to 2019 in Chinese Females Using Joinpoint Regression. ASDR: age-standardized disability-adjusted life-years rates; ASMR: age-standardized mortality rates.


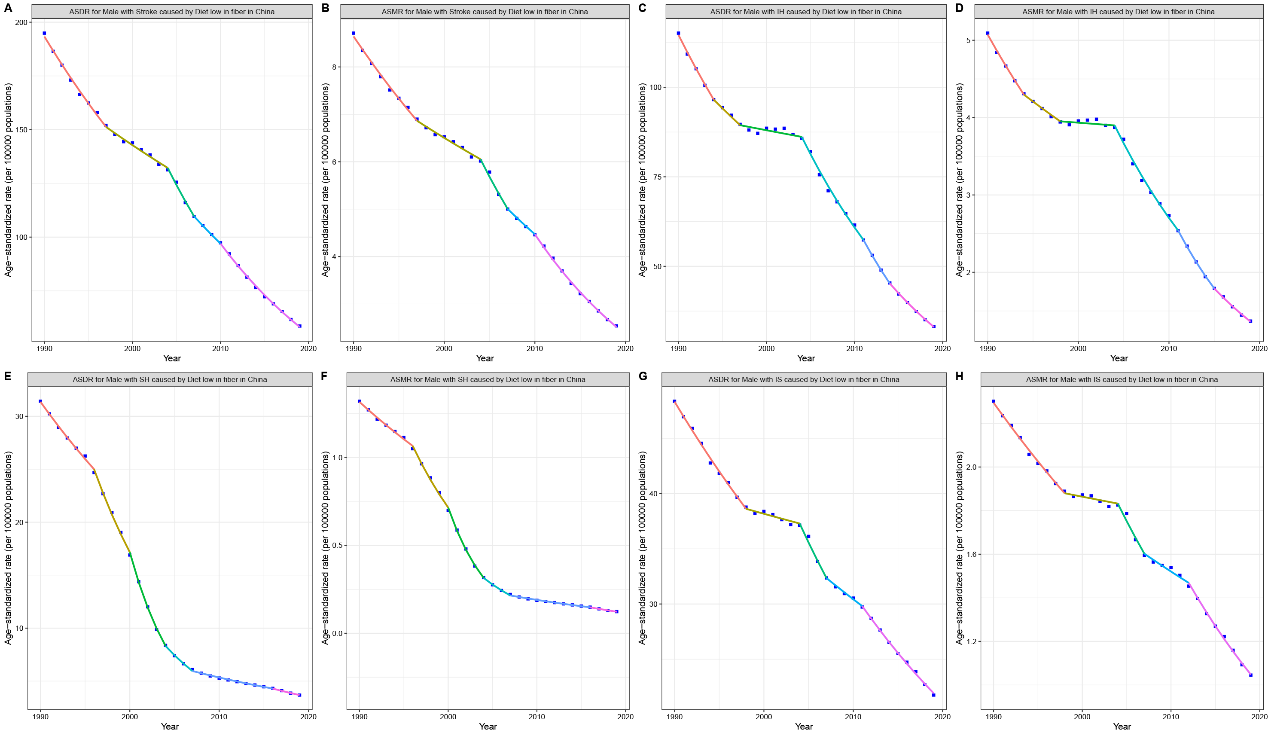


Figure S3. Trends of ASDR and ASMR of Stroke and its Subtypes Attributable to Low Dietary Fiber From 1990 to 2019 in Chinese Males Using Joinpoint Regression. ASDR: age-standardized disability-adjusted life-years rates; ASMR: age-standardized mortality rates.


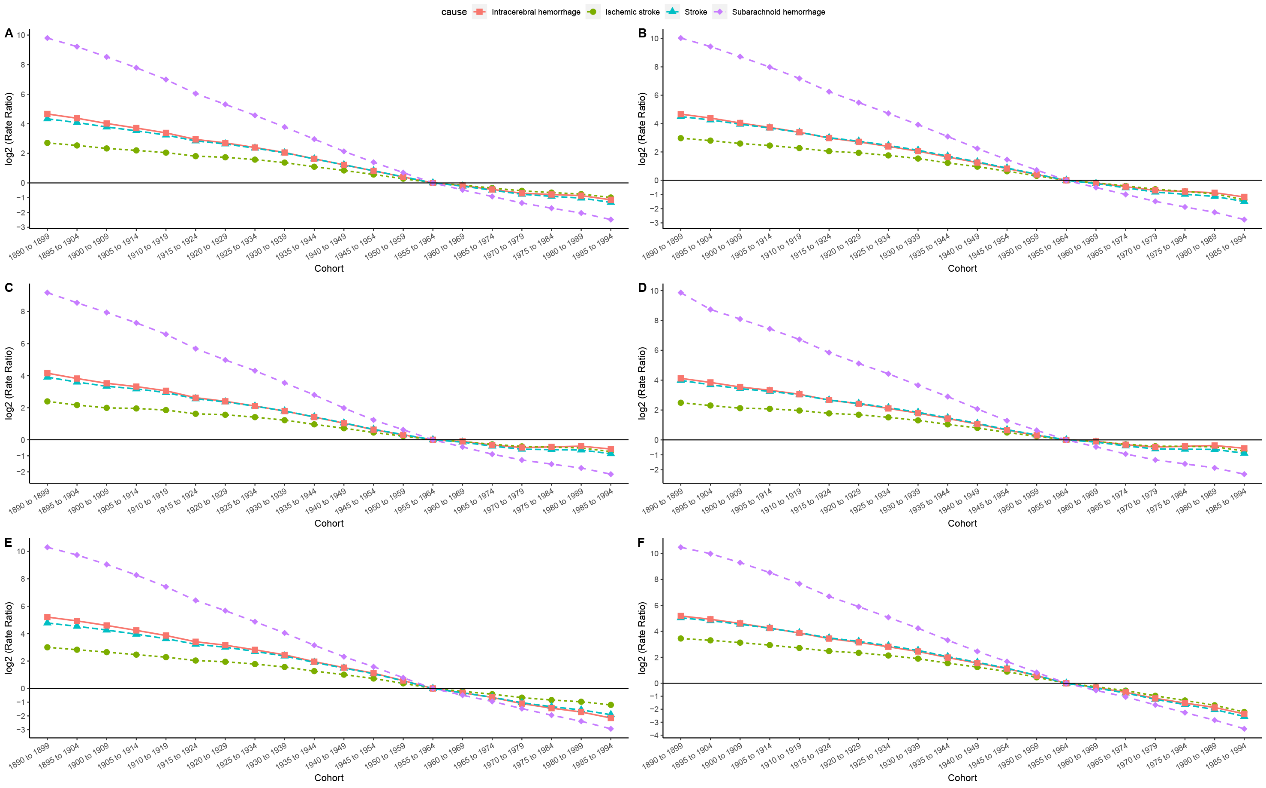


Figure S4. Cohort effects of stroke attributable to low fiber dietary intake and its subtypes ASDR and ASMR in China. (A) All ASDR, (B) male ASDR, (C) female ASDR, (D) all ASMR, (E) male ASMR, and (F) female ASMR. ASDR: age-standardized disability-adjusted life-years rates; ASMR: age-standardized mortality rates.
